# ^**18**^F-FDG PET/CT and MRI in the Management of Multiple Myeloma: A Comparative Review

**DOI:** 10.3389/fnume.2021.808627

**Published:** 2022-02-22

**Authors:** Charles Mesguich, Cyrille Hulin, Valérie Latrabe, Axelle Lascaux, Laurence Bordenave, Elif Hindié

**Affiliations:** ^1^Department of Nuclear Medicine, Centre Hospitalier Universitaire de Bordeaux, Bordeaux, France; ^2^University of Bordeaux, IMB, UMR CNRS 5251, INRIA Project Team Monc, Talence, France; ^3^Department of Haematology, Centre Hospitalier Universitaire de Bordeaux, Bordeaux, France; ^4^Department of Radiology, Centre Hospitalier Universitaire de Bordeaux, Bordeaux, France; ^5^University of Bordeaux, INCIA UMR-CNRS 5287, Talence, France; ^6^Institut Universitaire de France (IUF), Paris, France

**Keywords:** FDG-PET/CT, MRI, diffusion-weighted (DW) MRI, multiple myeloma, minimal residual disease (MRD)

## Abstract

During the last two decades, the imaging landscape of multiple myeloma (MM) has evolved with whole-body imaging techniques such as fluorodeoxyglucose positron emission tomography–computed tomography (^18^F-FDG PET/CT) and MRI replacing X-ray skeletal survey. Both imaging modalities have high diagnostic performance at the initial diagnosis of MM and are key players in the identification of patients needing treatment. Diffusion-weighted MRI has a high sensitivity for bone involvement, while ^18^F-FDG PET/CT baseline parameters carry a strong prognostic value. The advent of more efficient therapeutics, such as immunomodulatory drugs and proteasome inhibitors, has called for the use of sensitive imaging techniques for monitoring response to treatment. Diffusion-weighted MRI could improve the specificity of MRI for tumor response evaluation, but questions remain regarding its role as a prognostic factor. Performed at key time points of treatment in newly diagnosed MM patients, ^18^F-FDG PET/CT showed a strong association with relapse risk and survival. The deployment of minimal residual disease detection at the cellular or the molecular level may raise questions on the role of these imaging techniques, which will be addressed. This review summarizes and outlines the specificities and respective roles of MRI and ^18^F-FDG PET/CT in the management of MM.

## Introduction

Historically, multiple myeloma (MM) bone extent was assessed by a skeletal survey. This technique was part of the Durie–Salmon classification (1). Whole-body X-ray is, however, limited by its lack of sensitivity, partly because MM osteolytic lesions start being visible when more than 30% of the trabecular bone is involved (2).

Functional imaging with the use of fluorodeoxyglucose positron emission tomography–computed tomography (^18^F-FDG PET/CT) and MRI has progressively replaced skeletal survey. These whole-body imaging techniques had first been incorporated in the Durie and Salmon Plus classification (3). Both allow a more precise evaluation of tumor burden in bone/bone marrow, which has been recognized as an important prognostic factor in MM. Nowadays, bone involvement is defined by ^18^F-FDG PET/CT or MRI following the International Myeloma Working Group (IMWG) recommendations for MM (4). Both imaging modalities are therefore key players in the identification of myeloma patients needing treatment.

During the last decade, several studies have been published on the respective contribution of ^18^F-FDG PET/CT and MRI in the management of MM. Newer MRI techniques with the use of diffusion imaging have also emerged. The goal of this review was to summarize and outline the specificities and respective roles of both imaging modalities in MM.

## Role of Whole-Body Low-Dose Computed Tomography

Advances in technology now allow performing CT with a low radiation dose while preserving image quality. In 2005, Horger et al. introduced this technique for MM imaging, showing an excellent inter-observer agreement of 95% (5). Whole-body low-dose CT (WBLDCT) is a faster imaging procedure and is useful in evaluating the risk of spine fracture instability. Overall, when compared with skeletal survey, WBLDCT detects additional lesions in about 20% of patients, especially in the spine and pelvis (6–9). Performing WBLDCT at baseline can also lead to a change in the clinical management of 18–20% of patients (6–8). It is, however, limited on some points: the detection of extra-medullary lesions and the assessment of diffuse bone marrow infiltration (10).

WBLDCT is one of the recommended imaging procedures for baseline imaging of MM following the guidelines of IMWG, the European Society for Medical Oncology (ESMO), and the European Myeloma Network (11–13). The detection of ≥1 osteolytic focal lesion (FL) is a sufficient criterion for treatment initiation. Only bone lesions ≥5 mm should be looked at to avoid false-positive findings, considering the high frequency of osteoporosis in this population (10). Of note is that WBLDCT images can replace standard CT in PET/CT procedures without any noticeable degradation in the attenuation-corrected PET scan (14).

## Comparison of ^18^F-FDG PET/CT and MRI at Baseline Evaluation of MM

### Fundamental Concepts of Imaging Signal

^18^F-FDG is a biochemical analog of glucose. It is imported through the cell by GLUT-1 and GLUT-3 transporters and phosphorylated by a hexokinase, then becoming ^18^F-FDG-6P. Under this form, ^18^F-FDG-6P cannot be further metabolized through the Krebs cycle for the purpose of aerobic glycolysis. This metabolism dead-end contributes to the accumulation of ^18^F-FDG-6P in the cell, reaching a state of equilibrium within 60 min after 18-FDG administration. MM plasma cells generally have an overexpression of hexokinase-2 helping them reach a high glycolytic activity. Therefore, in MM, 18-FDG focal uptake on PET/CT is a reflection of packed MM cells with higher glycolytic activity than normal surrounding cells.

In the bone marrow, the signal intensity of MRI depends on the proportions of hematopoietic red marrow, fatty yellow marrow, and, to a lesser extent, mineralized bone matrix. T1-weighted images reflect bone marrow fatty content, as the protons contained in the heavy molecular hydrophobic complex have a very efficient spin–lattice relaxation, resulting in a short T1 relaxation time, i.e., a high T1-weighted signal. Short tau inversion recovery (STIR) T2-weighted (T2w-STIR) sequences perform a homogenous suppression of fat signals by exploiting the difference in the relaxation times between water and fat. Multiple Myeloma lesions have high cellularity and water content, therefore appearing as hypointense on T1- and hyperintense on T2w-STIR images.

Diffusion-weighted imaging studies the Brownian stochastic movement of water molecules within extracellular, intracellular, and intravascular spaces, allowing the study of the tissue microarchitecture. Water diffusion in biological tissues is restrained by cellular membranes, organites, and macromolecules. Therefore, diffusion can be decreased in tissues with high cellular density, intact cellular membranes, or reduced extracellular space. The apparent diffusion coefficient (ADC) is a quantitative biomarker of diffusion. In normal bone marrow, the diffusion signal is decreased in fatty marrow due to large adipocytes restraining the extracellular space and is increased in red marrow due to the higher vascularization and water content. Bone marrow infiltration by MM cells creates a high diffusion signal due to the replacement of yellow marrow adipocytes and to an increased cellular density.

### Disease Presentation and Imaging Patterns

Different imaging patterns of MM bone involvement have been described. Patients can present only with FLs, which are defined on ^18^F-FDG PET/CT as a focal uptake above bone marrow background with an underlying osteolytic lesion on companion CT (4). In numerous relevant studies, the definition of FL has been extended to focal bone uptake on two consecutive PET slices without evident changes on CT (15–17). On MRI, FL is defined as a lesion with a low T1-weighted signal and a high T2w-STIR signal (18). Two other patterns have been described: diffuse bone marrow infiltration (diffuse disease) only and the combination of FL and diffuse disease. Diffuse disease is typically described on MRI as a diffuse bone marrow hypointense signal on T1-weighted images associated with a diffuse bone marrow high signal on T2w-STIR images (19). The severity degree of diffuse disease can be further characterized into mild to moderate or severe depending on the intensity of signal modification on T1-weighted sequences by comparing it to the intervertebral disk signal (19). However, diffuse disease can sometimes be difficult to diagnose on MRI due to varying imaging features depending on the degree of infiltration. Diffusion-weighted MRI (DW-MRI) may help in that regard. On ^18^F-FDG PET/CT, diffuse disease diagnosis can be missed, especially in cases of mild and moderate infiltration due to low plasma cell density, unless the cell avidity for FDG is high. Diffuse bone marrow infiltration has been described as a diffuse bone marrow uptake superior to liver uptake (17, 20). An additional variegated pattern has been described on MRI and corresponds to multiple micronodular bone marrow lesions. Most authors consider this entity as a low-grade diffuse infiltration (21). The IMWG recommendations state that the presence of ≥1 osteolytic FL on ^18^F-FDG PET/CT or ≥2 FLs on MRI is a sufficient criterion to define bone involvement and start patient treatment (4).

Extramedullary disease (EMD) can be depicted by ^18^F-FDG PET/CT or whole-body MRI (22–24). It corresponds to disease sites located outside the bone structure and can be located in any organ or soft tissue (25). It should be differentiated from paramedullary disease (PMD), which corresponds to breakout bone lesions that invade the surrounding soft tissues. The two entities are different as EMD is composed of immature plasmablastic cells and PMD is made up of plasma cells (26). Approximatively 2–14% of patients have primary EMD at the initial diagnosis, and about 8–18% will develop EMD during the disease course throughout therapeutic sequences (26, 27). The most frequent sites involved are the pleura, liver, lymph nodes, spleen, subcutaneous tissue, and paravertebral areas (23, 28).

^18^F-FDG PET/CT readings can be standardized with the Italian Myeloma Criteria for PET Use (IMPETUS) (29, 30). The IMPETUS criteria have shown high inter-observer reproducibility (30). The main characteristics of both imaging modalities at baseline are summarized in Table 1.

**Table 1:** Main characteristics of ^18^F-FDG PET/CT and MRI for multiple myeloma imaging at baseline.

	**^**18**^F-FDG PET/CT**	**MRI**
Scanning time	15–20 min Starts 60 min after FDG injection	Between 30 and 50 min
Radiation exposure	10–25 mSv (PET+CT component)	None
Bone involvement	High sensitivity ~10% of PET false-negative MM	High sensitivity of AS-MRI Highest sensitivity of WB DW-MRI
Diffuse bone marrow disease	Moderate sensitivity	Gold standard
Extramedullary disease	Preferred technique	Diagnostic value less explored
Impact on clinical decision	More than WB DW-MRI	Less than ^18^F-FDG PET/CT
Prognostic value	>3FL EMD SUV_max_ Other quantitative PET parameters (MTV and TLG)	Diffuse disease >3 large FLs on WB DW-MRI (>5 cm^2^)
Standardization for acquisition, interpretation, and reporting	IMPETUS criteria	WB DW-MRI: MY-RADS criteria

^18^*F-FDG PET/CT, fluorodeoxyglucose positron emission tomography–computed tomography; MM, multiple myeloma; AS-MRI, axial skeleton MRI; WB DW-MRI, whole-body diffusion-weighted MRI, FL, focal lesion; MTV, metabolic tumor volume; TLG, total lesion glycolysis; SUV_max_, maximum standardized uptake value; IMPETUS, Italian Myeloma Criteria for PET Use; MY-RADS, Myeloma Response Assessment and Diagnosis System*.

### ^18^F-FDG PET/CT Compared With Conventional MRI for Baseline Evaluation

Earlier studies compared the detection rates of ^18^F-FDG PET/CT and axial skeleton (AS) MRI (spine/pelvis) in newly diagnosed MM (NDMM) patients (22, 31, 32). Overall, the results showed that ^18^F-FDG PET/CT and MRI have similar FL detection rates. It should be stressed that most of the additional lesions detected by ^18^F-FDG PET/CT were outside the field of view of AS-MRI. These studies also showed that MRI is superior to ^18^F-FDG PET/CT for the diagnosis of diffuse disease (22, 32, 33). However, a larger prospective study of 134 patients found no statistically significant difference in the concordance of AS-MRI and ^18^F-FDG PET/CT for the diagnosis of myeloma bone involvement (34). Compared to AS-MRI, whole-body MRI detects additional FLs located outside the axial skeleton (211 lesions in a study of 100 patients) (35). When compared with whole-body MRI, ^18^F-FDG PET/CT had a slightly lower sensitivity (59 vs. 68%) in a study of 24 patients (36). A recent study has shown that, although whole-body MRI had higher sensitivity than ^18^F-FDG PET/CT in 40 NDMM, ^18^F-FDG PET/CT had a higher impact on clinical decisions (37). Currently, ^18^F-FDG PET/CT or low-dose whole-body CT is recommended for patients with suspected myeloma as first-line imaging. MRI should be performed in the case of negative or inconclusive findings (11). Local availability and expertise may influence the choice of imaging modality.

### ^18^F-FDG PET/CT Compared With Diffusion-Weighted MRI for Baseline Evaluation

DW-MRI is a newer technique that complements standard MRI sequences. Diffusion imaging studies the diffusion of water molecules within extracellular, intracellular, and intravascular spaces (38). Diffusion is restrained in tissues with high cellular density, reduced extracellular space, and if cellular membranes are intact (39). It can further increase diagnostic confidence of FL and diffuse disease with the help of the ADC measurement (40, 41). A few studies compared the diagnostic performance of DW-MRI with ^18^F-FDG PET/CT in a pairwise fashion. A large study of 227 NDMM found that, in about 10% of patients, DW-MRI was positive, but with no apparent disease on ^18^F-FDG PET (42). In that study, data from companion CT of PET were not used to correct the false-negative PET cases. These patients likely have a lower expression of hexokinase-2, therefore hampering ^18^F-FDG PET evaluation (42). Similar results were found by two other recent studies (43, 44). It was also shown that CT helps improve the diagnosis of bone involvement in patients with a low ^18^F-FDG uptake (44, 45). Comparison of the detectability of FLs showed that, overall, DW-MRI detects more FLs than does ^18^F-FDG PET/CT (44–46). Reports agree that ^18^F-FDG PET/CT appears to be superior for the detection of upper limb FLs, while DW-MRI seems better at analyzing the skull, the spine, and the pelvis (44, 46). A prospective study found that, although DW-MRI detected more FLs compared to ^18^F-FDG PET/CT, there was no difference regarding bone disease diagnosis on a per patient basis, with an agreement of 1.0 (44). This goes against a retrospective study that reported an overall sensitivity for detecting bone disease on a per patient basis of 69.6% for ^18^F-FDG PET/CT vs. 91.3% for DW-MRI using an independent clinical reference standard (45). However, the authors reported that performing DW-MRI in addition to ^18^F-FDG PET/CT did not significantly change the clinical decision on treatment (45).

### Baseline Imaging Techniques as Prognostic Factors

The value of baseline ^18^F-FDG PET/CT as a prognostic factor has been demonstrated in historical cohorts, which consistently showed that FL maximum standardized uptake value (SUV_max_) (cutoff >3.9 or >4.2), the number of FLs (>3FL), and EMD are independently associated with progression-free survival (PFS) and overall survival (OS) (15, 16, 47). In populations of patients treated with the current standard of care comprising immunomodulatory drugs, recent works have shown that EMD and >3FL maintain a predictive value for PFS and OS (48–53). The threshold of FL SUV_max_ appears, however, to be higher (>6.3) in order to maintain a significant association with OS in patients treated with newer drugs, possibly due to the more effective treatments (48). More advanced imaging markers are also under study. Whole-body metabolic tumor volume (MTV) and whole-body tumor lesion glycolysis (TLG) (i.e., MTVxSUV_mean_) are promising prognostic markers showing superiority in comparison with the baseline number of FLs or baseline SUV_max_ in a cohort of patients treated with chemotherapy comprising thalidomide, dexamethasone, cisplatin, cyclophosphamide, and etoposide, followed by intensification by melphalan, tandem autologous stem cell transplant (ASCT), and maintenance by thalidomide and dexamethasone (total therapy 3 protocol) (54). Similar results were found in a study of 185 NDMM patients treated with a proteasome inhibitor-based regimen (55).

In MM patients treated with the total therapy 3 protocol, a study of 611 patients showed that >7FL on AS-MRI had a negative impact on OS (56). Furthermore, the combination of >7FL on MRI with cytogenetic abnormalities defined a group with a dismal prognosis (56). This FL threshold was found to be higher (>25 FLs) in a large study of patients undergoing whole-body MRI with similar treatments (57). Regarding diffuse bone marrow disease on MRI, several reports have found it to be associated with higher plasma cell infiltration, higher lactate dehydrogenase (LDH), higher incidence of anemia, and worse survival (47, 58, 59). Limited data are available regarding the predictive value of baseline DW-MRI parameters for PFS or OS. In 404 transplant-eligible patients undergoing baseline DW-MRI, Rasche et al. found that the presence of more than 3 large FLs (>5 cm^2^) was a strong independent prognostic factor for PFS and OS (60). Additionally, the number of FLs on MRI lost its predictive value for outcomes when adjusting the size of FLs (60). This underlines the importance of tumor burden as a prognostic factor. It was also found that a high tumor burden was associated with a lack of spleen signal on DW-MRI (61). Another smaller prospective study found that baseline FL ADC was not a significant prognostic factor (49).

The predictive value for relapse and survival of the two imaging techniques at baseline is summarized in Table 1.

## ^18^F-FDG PET/CT and MRI for Treatment Response Evaluation

The value of ^18^F-FDG PET/CT and MRI as prognostic factors has been studied at different time points of MM treatment. The main characteristics of both modalities in the evaluation of treatment response are summarized in Table 2.

**Table 2:** Main characteristics of ^18^F-FDG PET/CT and MRI for multiple myeloma evaluation of response to treatment.

	**^**18**^F-FDG PET/CT**	**MRI**
FL signal change in responders	Quick decrease of FL SUV_max_ (as soon as 7 days post-chemotherapy)	Conventional MRI: takes longer than ^18^F-FDG PET/CT to change DW-MRI: elevation of ADC as soon as 3 weeks post-chemotherapy
Standardization of interpretation criteria	After ASCT CMR: FL and BM uptake less than the liver uptake	MY-RADS criteria
Post-induction chemotherapy prognostic value	Variable prognostic value	Not statistically significant
Post-ASCT prognostic value	Prognostic factor for PFS and OS	MRI: No significant prognostic value DW-MRI less than ^18^F-FDG PET/CT
MRD	Complementary to molecular MRD (NGS or MFC). Defines imaging MRD subgroups	Unknown value

^18^*F-FDG PET/CT, fluorodeoxyglucose positron emission tomography–computed tomography; FL, focal lesion; DW-MRI, diffusion-weighted MRI; ADC, apparent diffusion coefficient; ASCT, autologous stem cell transplant; CMR, complete metabolic response; BM, bone marrow; MRD, minimal residual disease; NGS, next-generation sequencing; MFC, multiparameter flow cytometry; SUV_max_, maximum standardized uptake value; MY-RADS, Myeloma Response Assessment and Diagnosis System*.

### ^18^F-FDG PET/CT Compared With Conventional MRI After Induction Chemotherapy

The team from the Bologna Center showed that SUV_max_ > 4.2 on ^18^F-FDG PET/CT performed after induction chemotherapy had a negative impact on the PFS of 85 patients treated with tandem transplant (16). These results slightly differed from those provided by the Little Rock (Arkansas) Center, which showed that the disappearance of FLs on pre-transplant ^18^F-FDG PET/CT had a positive impact on the PFS and OS of patients treated using the total therapy 3 protocol (51).

The multicenter IMAJEM Study considered post-induction ^18^F-FDG PET/CT as negative if the FL uptake was inferior or equal to the liver uptake in a mixed population of 134 patients treated either with a bortezomib-based chemotherapy (*N* = 71) followed by maintenance or with induction chemotherapy followed by ASCT, consolidation, and maintenance chemotherapy. The authors found no significant impact of the post-induction ^18^F-FDG PET/CT results on PFS and OS (17).

However, in a prospective study of 30 patients all treated with an immunomodulatory-based induction chemotherapy followed by ASCT and consolidation, post-induction ^18^F-FDG PET/CT showed a significant association with PFS with the same positivity threshold as that used in the IMAJEM Study (49).

Quantifying the variations in SUV_max_ is another interesting approach. In the 71 patients in the IMAJEM Study with a baseline uptake superior to that of the liver, a decrease of more than 25% of the SUV_max_ after induction chemotherapy was associated with a benefit in PFS, which was superior to that obtained with the biochemical evaluation of response (62).

The value of MRI after induction chemotherapy has mainly been studied in pairwise comparisons with ^18^F-FDG PET/CT. In a prospective study of 332 patients, only 64 patients had a negative MRI after induction chemotherapy, whereas 245 had a negative ^18^F-FDG PET/CT. Contrary to ^18^F-FDG PET/CT, post-induction MRI had no significant association with PFS or OS (47). The IMAJEM Study found that, in 134 patients, only 3% had a negative MRI after induction chemotherapy, whereas 32% had a negative ^18^F-FDG PET/CT (17). Similarly, there was no association of the MRI results with PFS or OS (17). These results illustrate the lack of specificity of the MRI signal after induction chemotherapy.

### ^18^F-FDG PET/CT Compared With Conventional MRI After ASCT

In patients treated with thalidomide and tandem ASCT, the Bologna team showed that a complete decrease of the FL SUV_max_ on post-transplant ^18^F-FDG PET/CT had a significant association with PFS and OS (16). The authors also found that all patients who had an SUV_max_ > 4.2 on post-ASCT ^18^F-FDG PET/CT relapsed during follow-up (63). Similarly, the team from Little Rock showed that a 100% decrease of FL uptake was associated with better PFS and OS, with prognosis similar to that of patients without FL at baseline (51).

The IMAJEM Study, using the liver SUV_max_ as the threshold for ^18^F-FDG PET/CT positivity, found that pre-maintenance ^18^F-FDG PET/CT had a strong association with PFS and OS as well, but only in the group of patients who underwent ASCT (17). Conversely, pre-maintenance MRI had no significant association with relapse or survival (17). The FL uptake of ^18^F-FDG PET/CT quickly changes after the start of MM treatment, whereas the FL signal on MRI will take longer to normalize after treatment (32, 64). In some cases, liquid transformation of focal lesions can occur, with an increase in the T2-weighted and DW imaging signals and in ADC, which will persist indefinitely.

A joint French–Italian cohort of transplant-eligible MM patients from the IFM/DFCI2009 and EMN02/HO95 studies confirmed the value of pre-maintenance ^18^F-FDG PET/CT as a prognostic factor for PFS and OS (65). This study also provided standardized criteria for post-ASCT response evaluation, further refining the IMPETUS criteria, with a complete metabolic response defined as a focal and/or diffuse bone marrow uptake lower than the liver background (30, 65).

Therefore, ^18^F-FDG PET/CT is recommended over MRI as it provides an earlier evaluation of the treatment response (66).

### ^18^F-FDG PET/CT Compared With Diffusion-Weighted MRI After ASCT

A few studies correlated the biochemical response of MM patients with DW-MRI ADC measurements (67–72). Different timings were used: either early (at 3 or 8 weeks post-chemotherapy) or later in the course of treatment (between 13 and 21 weeks post-chemotherapy). It is thought that an increase in the ADC would reflect tumor necrosis, microbleeding, tumor edema, and a decrease in cellular density (70). Conversely, a late evaluation would show a decrease in the ADC due to necrosis replacement by fatty bone marrow. Therefore, DW sequences could increase the specificity of MRI by providing a more dynamic evaluation of the FL response. Overall, studies agree that the ADC of FLs increases in patients with biochemical response, and thus at least until 21 weeks post-chemotherapy, whereas it is more uncertain in patients with diffuse bone marrow disease (68, 70–72). However, these studies suffer from a lack of harmonization regarding the time of MRI performance, the study population (newly diagnosed or treated MM), or the choice of *b* values for diffusion sequences. The Myeloma Response Assessment and Diagnosis System (MY-RADS) criteria have been recently published to address this need for harmonization by providing a classification of the probability of response or progression based on ADC measurements combined with morphological appreciation (73). These differed from the IMPETUS criteria, which provided a dichotomized classification of responses, individualizing high-risk patients.

Although the predictive value of ^18^F-FDG PET/CT on relapse and survival performed during treatment has been addressed, little is known about that of DW-MRI. A prospective study of 30 NDMM patients provided a head-to-head comparison of ^18^F-FDG PET/CT and DW-MRI performed after induction chemotherapy and after ASCT (49). ^18^F-FDG PET/CT was considered negative if the FL uptake was inferior to the liver uptake, and the DW-MRI response was addressed using the MY-RADS criteria (49). While ^18^F-FDG PET/CT showed a significant association with PFS at both times of treatment response evaluation, DW-MRI showed no value as a prognostic factor, within the limit of a small population (49). Similarly, another larger study comparing ^18^F-FDG PET/CT and DW-MRI without standardized reading criteria showed that the predictive value of ^18^F-FDG PET/CT on relapse was superior to that of DW-MRI (74). Larger standardized prospective studies are, however, warranted to confirm these results. Additionally, the complementary role of ^18^F-FDG PET/CT and DW-MRI should be further addressed, for example through studies performed on PET/MRI hybrid systems (75).

### Functional Imaging Complementarity With Biological MRD

Biological minimal residual disease (MRD) evaluation refers to highly sensitive techniques such as multiparameter flow cytometry (MFC) or next-generation sequencing (NGS), with a ratio of detection of 1 clonal cell in 10^5^–10^6^ normal cells on bone marrow biopsy (67–70). MRD evaluation is standardized by IMWG recommendations, which defined a new category of response depending on the results of MRD among patients with biochemical complete remission (CR) (76). In the IFM 2009 study, patients with biochemical CR and positive MRD by NGS had a 3-year PFS of 42 vs. 87% in patients with CR and negative MRD (77).

These highly sensitive molecular techniques can raise the question of the utility of imaging procedures. To address this matter, the IMAJEM Study showed that the concordance of pre-maintenance ^18^F-FDG PET/CT with the MRD results was low. In a group of 86 patients, ^18^F-FDG PET/CT and MRD double-negative patients had a 3-year PFS of 86.8% compared to 52.9% in patients with either ^18^F-FDG PET/CT and/or positive MRD (17). The CASSIOPET Study also found in 176 NDMM patients eligible for transplant that the concordance between MRD and post-consolidation ^18^F-FDG PET/CT was low (78). This illustrates the complementarity of both approaches. Indeed, MRD may be negative in patients with tumor clones outside the field of bone marrow sample either in the case of FLs outside the pelvic area or in case of EMD. Still, the frequent relapses of MRD-negative patients with CR support the idea of MRD persisting outside the pelvis (79). Evaluation of the role of potentially more sensitive and specific PET tracers in the detection of MRD and for patient prognostication is also awaited. Pilot observational studies using ^18^F-choline, ^11^C-methionine, ^11^C-acetate, or immuno-PET tracers targeting CD38 have provided the first steps to promising results (80–83). Studies evaluating the concordance of DW-MRI and biological MRD are also warranted.

## Conclusion

Whole-body functional imaging performed with ^18^F-FDG PET/CT or DW-MRI is a key player in the identification of NDMM patients needing treatment. The sensitivity of DW-MRI is superior to that of ^18^F-FDG PET/CT for the detection of bone involvement, and both modalities are crucial to detecting EMD and PMD (Table 1). Imaging modalities and reading are now standardized for both of these techniques, potentially adding to their clinical robustness. During treatment, the value of DW-MRI as a prognostic factor appears to be lower than that of ^18^F-FDG PET/CT, but larger prospective studies are warranted on this matter. ^18^F-FDG PET/CT remains the imaging modality of choice for monitoring treatment response, especially in patients undergoing ASCT, defining imaging-MRD that complements molecular/cellular MRD measurements (Table 2). The strengths of ^18^F-FDG PET/CT lie in its relative simplicity of interpretation and quantification, while the complexity of MRI leaves room for variability in its interpretation and quantification, therefore requiring longer training and strict harmonization. Future studies will have to further address the complementarity of DW-MRI and ^18^F-FDG PET/CT during the treatment of MM patients. Trials integrating imaging MRD measurements with ^18^F-FDG PET/CT for treatment decisions will be needed to further assess its prognostic value. This review mainly focused on the initial management of NDMM patients. Fewer studies considering their respective roles in the first and subsequent relapses are available. Again, more studies are needed to address the role of novel therapies involving antibodies, antibody–drug conjugates, bispecific antibodies, and chimeric antigen receptor (CAR) T cells. The possible roles of new promising PET tracers in the improvement of MRD assessment will also have to be addressed.

## Author Contributions

CM and EH wrote the manuscript. All authors contributed to the article and approved the submitted version.

## Conflict of Interest

The authors declare that the research was conducted in the absence of any commercial or financial relationships that could be construed as a potential conflict of interest.

## Publisher's Note

All claims expressed in this article are solely those of the authors and do not necessarily represent those of their affiliated organizations, or those of the publisher, the editors and the reviewers. Any product that may be evaluated in this article, or claim that may be made by its manufacturer, is not guaranteed or endorsed by the publisher.
